# Thermal Degradation Kinetics and Modeling Study of Ultra High Molecular Weight Polyethylene (UHMWP)/Graphene Nanocomposite

**DOI:** 10.3390/molecules26061597

**Published:** 2021-03-13

**Authors:** Iman Jafari, Mohamadreza Shakiba, Fatemeh Khosravi, Seeram Ramakrishna, Ehsan Abasi, Ying Shen Teo, Mohammadreza Kalaee, Majid Abdouss, Ahmad Ramazani S. A, Omid Moradi, Erfan Rezvani Ghomi

**Affiliations:** 1 Department of Civil and Environmental Engineering, Faculty of Engineering, National University of Singapore, Singapore 117576, Singapore; iman.jafari@u.nus.edu (I.J.); yingshen@u.nus.edu (Y.S.T.); 2 Department of Chemistry, Amirkabir University of Technology, Tehran 15875-4413, Iran; rezashakiba011@gmail.com (M.S.); phdabdouss44@aut.ac.ir (M.A.); 3 Department of Mechanical Engineering, Center for Nanofibers and Nanotechnology, Faculty of Engineering, National University of Singapore, Singapore 117581, Singapore; fatemeh_khosravi22@yahoo.com; 4 Department of Polymer and Chemical Engineering, South Tehran Branch, Islamic Azad University, Tehran 17776-13651, Iran; abasiehsan@ymail.com; 5 Nanotechnology Research Centre, Tehran South Branch, Islamic Azad University, Tehran 15847-43311, Iran; 6 Department of Chemical and Petroleum Engineering, Sharif University of Technology, Tehran 11365-9465, Iran; ramazani@sharif.edu; 7 Department of Chemistry, Shahre-Qods Branch, Islamic Azad University, Shahre-Qods 37515-374, Iran; moradei.omid@gmail.com

**Keywords:** ultra-high molecular weight polyethylene, graphene, thermal properties, nanocomposite, thermal degradation, modeling

## Abstract

The incorporation of nanofillers such as graphene into polymers has shown significant improvements in mechanical characteristics, thermal stability, and conductivity of resulting polymeric nanocomposites. To this aim, the influence of incorporation of graphene nanosheets into ultra-high molecular weight polyethylene (UHMWPE) on the thermal behavior and degradation kinetics of UHMWPE/graphene nanocomposites was investigated. Scanning electron microscopy (SEM) analysis revealed that graphene nanosheets were uniformly spread throughout the UHMWPE’s molecular chains. X-Ray Diffraction (XRD) data posited that the morphology of dispersed graphene sheets in UHMWPE was exfoliated. Non-isothermal differential scanning calorimetry (DSC) studies identified a more pronounced increase in melting temperatures and latent heat of fusions in nanocomposites compared to UHMWPE at lower concentrations of graphene. Thermogravimetric analysis (TGA) and derivative thermogravimetric (DTG) revealed that UHMWPE’s thermal stability has been improved via incorporating graphene nanosheets. Further, degradation kinetics of neat polymer and nanocomposites have been modeled using equations such as Friedman, Ozawa–Flynn–Wall (OFW), Kissinger, and Augis and Bennett’s. The "Model-Fitting Method” showed that the auto-catalytic nth-order mechanism provided a highly consistent and appropriate fit to describe the degradation mechanism of UHMWPE and its graphene nanocomposites. In addition, the calculated activation energy (E_a_) of thermal degradation was enhanced by an increase in graphene concentration up to 2.1 wt.%, followed by a decrease in higher graphene content.

## 1. Introduction 

Ultra-high molecular weight polyethylene (UHMWPE) is renowned for its stellar mechanical properties, high abrasion resistance, low moisture absorption, low friction coefficient, and excellent chemical stability [1,2]. UHMWPE is used to produce bullet-proof vests in climbing equipment, including ropes, fishing nets in the fisheries industry, and artificial bone in medical science [1,3,4]. Recently, several studies have been reported to enhance both the mechanical and thermal characteristics of UHMWPE using different types of nanofillers [5,6,7]. By incorporating the functional inorganic fillers such as graphene nanosheets in UHMWPE, some important properties of UHMWPE can be significantly improved such as wear resistance, stiffness, and deformation heat-resistance [8]. This is due to the unique properties of graphene such as high electrical, thermal, and mechanical properties [9]. This modification in the structure of UHMWPE can also decrease its gas permeability and flammability, and give it new functional properties [8]. 

The final properties of nanocomposites generally depended on the nature of the nanofillers and how they were fabricated. The main problem with the implementation of melt intercalation and solution casting methods for UHMWPE composite preparation is its extra high viscosity and lack of flowability in the melted UHMWPE and its solution. The in situ polymerization method is, by far, the best method for dispersing fillers for more efficient contact between the filler and the polymer matrix [10,11]. The above-mentioned method [3,9] helps prevent nanoparticles from aggregating and thus enhances the compatibility between nanoparticles and UHMWPE by supporting a suitable catalyst on nanoparticles’ surface. The metallocene can be used as an effective polymerization catalyst for the in situ preparation of UHMWPE/multiwalled carbon nanotube (MWCNT) and polypropylene/MWCNT nanocomposites [12,13]. Graphene is a material with extreme potential due to its multifaceted and multifarious properties, thus enabling other graphite derivatives to be used as a filler for many potential applications [14,15,16,17,18,19]. 

In general, graphene oxide (GO) is synthesized by methods developed by Brodie, Staudenmaier, and Hummers, or either of the above three with some permutation to the methodology [20]. In these methods, graphite is oxidized to produce appropriate GO, and then GO is reduced to graphene. In the Hummers method, graphite is oxidized via potassium permanganate (KMnO_4_) and sulfuric acid (H_2_SO_4_), while in the Brodie method, potassium chlorate (KClO_3_), and nitric acid (HNO_3_) are used to oxidize graphite. Staudenmaier’s method also uses a procedure like Brodie’s to oxidize graphite to GO. Stürzel et al. [21] applied polymerization filling techniques (PFT) to hone the compatibility between graphene and UHMWPE via a “single-site catalyst” supported by dispersed functionalized graphene nanosheets. In some other studies, graphene nanosheets have also been used to hone some polymeric properties [22,23,24].

Out of many analytical methods, thermogravimetric analysis (TGA) is the most preferred for assessing the influence of nanoparticles on the thermal degradation of polymers [25,26]. For instance, to evaluate the correlation between the effects of nanofillers on the temperature of degradation and the E_a_ of nanocomposites, compared to those resulting from the degradation of pure polymers [1,27]. Much work has been conducted on investigating the thermal degradation kinetics of polyethylene (PE), its nanocomposites, various kinetic parameters, and the apparent activation energies for the degradation of high density polyethylene (HDPE)/SiO_2_ nanocomposites. It is widely agreed that that the incorporation of SiO_2_ nanoparticles improves the thermal stability of HDPE [28]. 

However, it has been also noted that nanoclay involvement has two contradictory effects on the thermal stability of polymer/clay nanocomposites in PE/clay nanocomposites produced by melt mixing and in situ polymerization [29,30]. Chrissafisa et al. have shown that nanocomposites containing 1 to 3 wt.% of organoclay are more thermally stable than neat polymer and that stability increases with the addition of organoclay [30]. Their results also revealed a decrease in thermal stability when the organoclay content reaches 5 wt.%. Furthermore, the thermal stability and activation energies were improved in UHMWPE/MWCNT nanocomposites by addition of MWCNT up to 1.5 wt.%. Moreover, the addition of 3.5 wt.% MWCNT decreased the activation energy of UHMWPE/MWCNT nanocomposite [21].

In the previous work by Shafiee and Ramazani, preparation of UHMWPE/graphene nanocomposites was reported using in situ polymerization, which could improve the compatibility between polymers and graphene and reduce the aggregation of graphene nanosheet in UHMWPE matrix [31]. This work aims to study the thermal behavior and degradation kinetics of in situ prepared UHMWPE/graphene nanocomposites by five different modeling methods and compare them as a novel study. Thermal properties such as melting point and thermal degradation of UHMWPE/graphene nanocomposites have been studied in the first part of the present work using various kinetic parameters such as initial degradation temperature (T_0.1_), decomposition temperature at 50% weight loss (T_0.5_), degradation temperature at maximum weight loss rate (T_m_), and the residual yields (W_R_) from TGA. Subsequently, models such as Friedman, Ozawa–Flynn–Wall (OFW), Kissinger, and the Augis and Bennett methods were also used to approximate the degradation activation energies of neat UHMWPE and its nanocomposites.

## 2. Materials and Methods

### 2.1. Materials

UHMWPE with a molecular weight of about $3\times 10^{6}g/\mathrm{mol}$ was prepared using in situ polymerization of ethylene with a Ziegler–Natta catalyst [31]. The graphite powder was purchased from the Dae-Jung Chemicals and Metals Co. (Siheung, Republic of Korea). Magnesium ethoxide (C_4_H_10_MgO_2_, 98%) was purchased from Fluka (Buchs, Switzerland). Triethyl aluminum (C_6_H_15_Al, 93%), N-hexane (≥99%), toluene (99.8%), ethylene (C_2_H_4_, ≥99.5%), titanium tetrachloride (TiCl_4_, ≥99%), dibutyl phthalate (99%), and anhydrous toluene (C_6_H_5_CH_3_, 99.8%) were purchased from Sigma–Aldrich (St. Louis, Missouri, MO, USA).

### 2.2. Fabrication of UHMWPE/Graphene Nanocomposites

The advanced Bi-supported Ziegler–Natta catalytic systems were used to prepare UHMWPE/graphene nanocomposites by in situ polymerization [31,32]. In the first step, graphite powder was vacuum-dried for 6 h at 200 °C. Oxidation of natural graphite flakes and preparation of graphene nanosheets were conducted through the modified Hummers method [33]. The most common method to modify graphite is the use of mineral acid which imparts acidity to the component. To this aim, graphite oxide and magnesium ethoxide in a weight ratio of 4:1 were added to a triple-necked reactor equipped with a vacuum pump connector, ethylene monomer cylinder, and a rubber septum for addition of other materials. A combination of *n*-hexane with toluene (150 mL) in 1:1 weight ratio was added to the reactor. The reactor was placed in an oil bath on a heater stirrer while the temperature was set on 80 °C. At 80 °C, the polymerization catalyst, TiCl_4_ (8 mL) in the form of the slurry, along with 1 mL dibutyl phthalate, were then moved to the polymerization reactor and stirred for 2 h. Then, the reactor was cooled down and graphene-catalyst complex was obtained after rinsing with *n*-hexane to remove the unreacted materials. Eventually, 100 mL of graphene-catalyst dissolved in *n*-hexane was used as a solvent to the graphene-catalyst and then was stored. 

In the next step, another reactor equipped with a mechanical stirrer, thermometer, and pressure controller was used for the preparation of UHMWPE/graphene nanocomposites. Firstly, 400 mL degassed *n*-hexane was added to the reactor. Afterward, triethyl aluminum (TEA) and then 10 mL graphene-catalyst complex (prepared in the previous step) were introduced to the system. Quickly, ethylene monomer was injected to the system at different concentrations to start polymerization of UHMWPE/graphene nanocomposites containing 0.9, 2.1, and 3.4 wt.% of graphene. To complete the polymerization after the desired time, 10 mL HCl was added to the reactor. Next, the reactor cooled down to the ambient temperature and products were also washed with ethanol, accompanied by filtration and vacuum-drying at 70 °C for 24 h. 

### 2.3. Morphological Characterization

The scanning electron microscopy (SEM) analysis was carried out by a TESCAN VEGA II (Brno, Czech Republic) model apparatus. Prior to imaging, the synthesized nanocomposites were cryogenically fractured in liquid nitrogen and then coated using the gold vapor deposition process using a K450X model vacuum sputter developed by EMITECH Co. 

The structure and degree of exfoliation of graphene layers in the prepared nanocomposites were investigated by a Philips X’pert Wide-angle X-Ray Diffraction (WXRD) system (Almelo, Netherlands) (40 kw, 30 mA). The spacing of the gallery was achieved according to Bragg’s Equation:

d= λ/2sin θ(1)

where d is the distance between graphene layers, λ is the X-ray wavelength, which is equal to 0.154 nm, and θ is the angle in the spectrum at the first peak.

### 2.4. Thermal Assessment

UHMWPE and its graphene nanocomposites were studied using thermogravimetric analysis (TGA) and differential scanning calorimetry (DSC), carried out using a Mettler TGA/SDTA 851e (Columbus, Ohio, OH, USA) instrument. Samples (about 5 mg) were heated from room temperature to 700 °C in N_2_ atmosphere at heating rates of 10, 15, and 20 °C min. Mettler Toledo Star Software was used for evaluating the data. The experiments were repeated three times and the average values, the best fit, and the standard deviations of parameters were provided.

### 2.5. Analytical Methods

The degradation mechanisms are sometimes unknown or very much complicated to understand via a simple kinetic model. A single step approximation method is widely used to understand the kinetics of thermal degradation, but it is also found that model-free (isoconversion method) or model-fitting approaches are viable [21,34]. Usually, the conversion rate for a solid-state reaction is presumed to be the multiplication of two parameters—the temperature (T) and a conversion function dependent on the extent of conversion of the reactant to products (α) [35]:

(2) $$\frac{d\alpha }{\mathrm{dt}}=k\left(T\right)f\left(\alpha \right)$$

where α is the degree of conversion, f(α) is the conversion function (reaction model), and k(T) is a temperature-dependent rate constant given by the Arrhenius Equation:

(3) $$k\left(T\right)=A\;\exp\left(\frac{-E_{a}}{\mathrm{RT}}\right)$$

Equation (2) can be rewritten as:

(4) $$\frac{d\alpha }{\mathrm{dt}}=\beta \frac{d\alpha }{\mathrm{dT}}=\mathrm{Af}\left(\alpha \right)\;\exp\left(\frac{-E_{a}}{\mathrm{RT}}\right)$$

where A, R, and E_a_ are the pre-exponential factor, the universal gas constant, and activation energy, respectively, and $\;\beta =\frac{\mathrm{dT}}{\mathrm{dt}}\;$= const is the linear heating rate in °C/min [1,21]. The analytical output should prepare an appropriate measurement with different temperature profiles by using a common kinetic model. The first step in the kinetic analysis is to evaluate the kinetic triplet parameters (conversion function (f(α)), E_a_, and pre-exponential factor (A)). From the mass curves reported in the TGA dynamic thermographs, the relation between conversion (α) and the kinetic parameters can be calculated. 

## 3. Results and Discussion

### 3.1. Morphology

**Figure 1:**
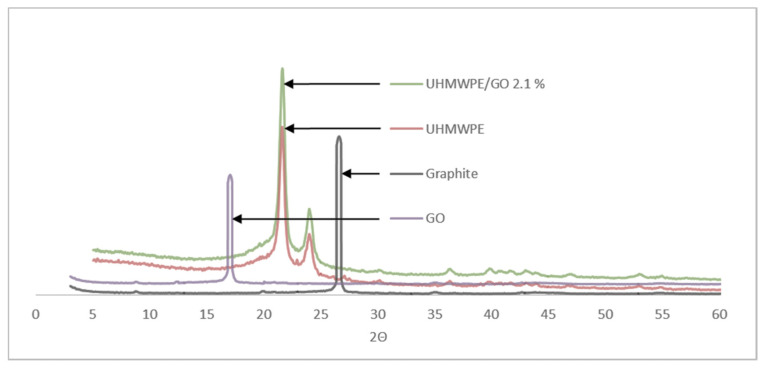
Graphite, graphene oxide, pure ultra-high molecular weight polyethylene (UHMWPE), and UHMWPE/graphene nanocomposite X-Ray Diffraction (XRD) patterns.

Prepared nanocomposites have been studied by XRD to determine the propensity of intercalation or exfoliation. The XRD patterns of the graphite, graphene oxide, pure UHMWPE, and UHMWPE/graphene nanocomposite with 2.1 wt.% are presented in Figure 1. The pristine graphite shows a basal reflection peak at 2θ = 26.6 corresponding to an interlayer spacing of 0.335 nm (Bragg’s Equation) and a crystalline peak for GO, which was not seen in the nanocomposite at 2θ = 17, showing the complete conversion of GO particles into the graphene sheets. Two sharp peaks at 2θ = 21.5 and 2θ = 24 were observed in UHMWPE and UHMWPE/graphene samples. As seen in Figure 1, there are no graphite or graphene oxide peaks in the nanocomposite curve, which indicates that GO is well exfoliated. Because XRD spectrums of different samples are taken in the almost same condition, comparison of spectrums of virgin and nanocomposite containing 2.1% nanographene reveals that crystallinity of UHMWPE could be increased in the presence of graphene. This could be due to the nucleating action of the nanofillers in the polymer matrix [36]. 

**Figure 2:**
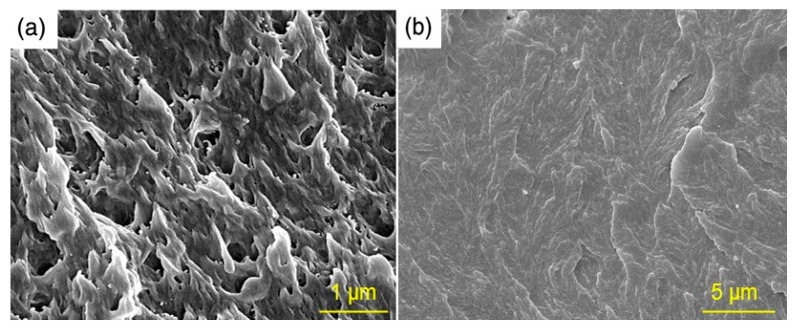
Scanning electron microscope (SEM) image of the fracture surface of (**a**) neat UHMWPE [37], and (**b**) UHMWPE/graphene 2.1% nanocomposite.

The SEM images of the fracture surface of neat UHMWPE and the nanocomposite with 2.1 wt.% of graphene loading are shown in Figure 2. Comparing Figure 2a,b, it can be seen that a homogeneous dispersion of graphene nanosheets was achieved in the polymer matrix and no agglomeration of graphene on the nanocomposite fracture surface was detected.

### 3.2. Differential Scanning Calorimetry (DSC)

To determine the melting temperature (T_m_) and latent heat of fusion (DH_f_) of pure UHMWPE and UHMWPE/graphene nanocomposites, differential scanning calorimetry (DSC) measurements were carried out and are shown in Table 1. 

**Figure 3:**
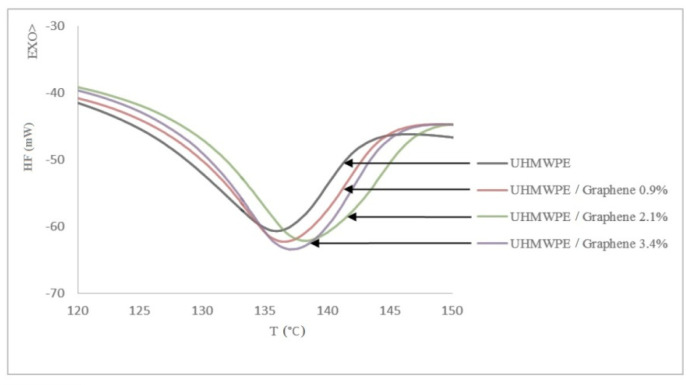
DSC heating of UHMWPE and UHMWPE/graphene nanocomposites.

The DSC heating for all samples is presented in Figure 3. The figure shows that the melting temperature and heat of fusion of samples increased upon the addition of 0.9 and 2.1 wt.% of graphene—however, the addition of 3.4 wt.% graphene decreased the above-mentioned values due to the agglomeration. Similar observations were reported in [3]. Generally, the addition of the filler at a lower concentration promotes crystallization to the semi-crystalline polymer matrix. Once the filler concentration extends beyond a certain threshold, there will be detrimental effects to the polymer matrix due to agglomeration.

### 3.3. Thermal Degradation Studies

**Figure 4:**
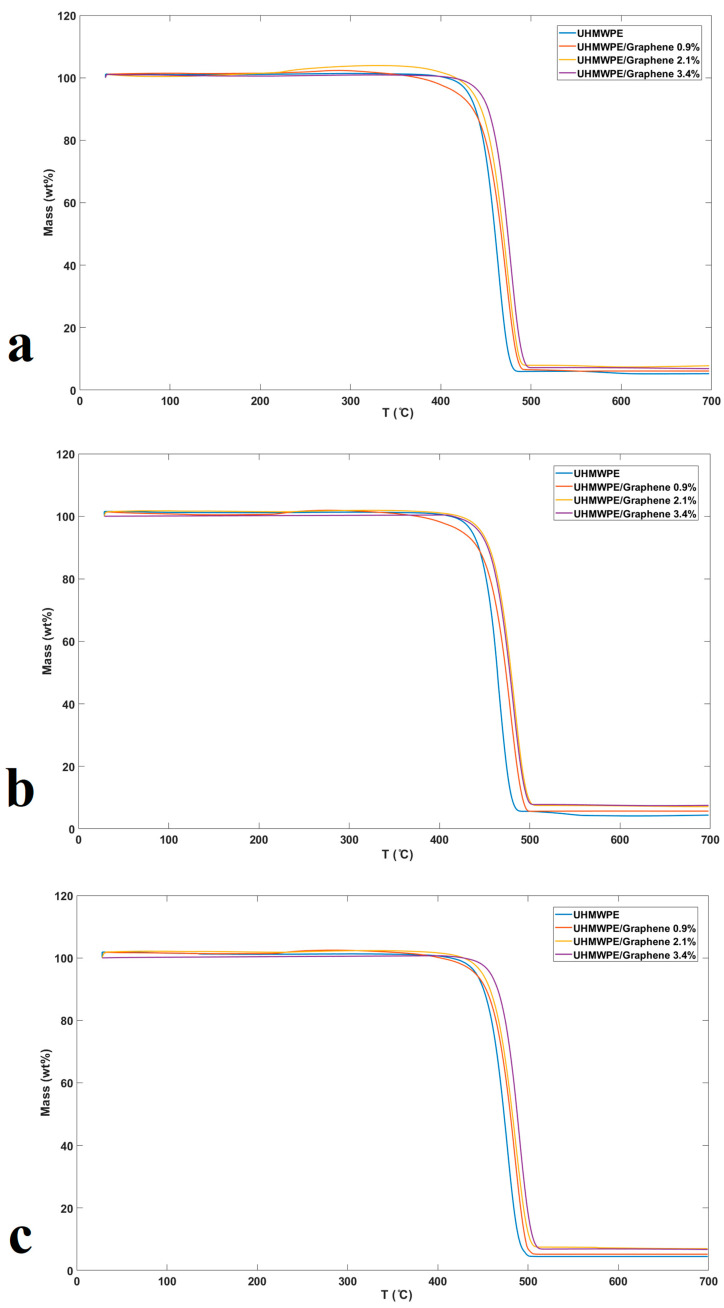
Mass loss (%) thermographs of pure UHMWPE and UHMWPE/graphene nanocomposites (0.9, 2.1, and 3.4% wt.) samples versus temperature at (**a**) 10 °C/min, (**b**) 15 °C/min, and (**c**) 20 °C/min.

The TGA analysis was used to investigate the thermal degradation of UHMWPE and UHMWPE/graphene nanocomposites. Figure 4a–c shows the thermographs of pure UHMWPE and UHMWPE/graphene nanocomposites at heating rates of 10 °C/min, 15 °C/min, and 20 °C/min. 

UHMWPE and UHMWPE/graphene nanocomposites are reported to have been thermally stable without weight loss of up to 370 °C. TGA parameters of UHMWPE and its graphene nanocomposites are presented in Table 2. Degradation temperatures of UHMWPE/graphene nanocomposites, including T_0.1_, T_0.5_, and T_m_, were increased with an increase in graphene content, demonstrating an increase in nanocomposite thermal stability compared to neat UHMWPE. Similar observations were reported in polystyrene composites and polyaniline nanocomposites [23,24,38]. The peak of the first derivative of mass loss (DTG) denotes T_m_, which is the degradation temperature at the maximum degradation rate. The polymerization of ethylene is actually supported by the presence of a catalyst on the graphene surface, and as reported, PE is covalently bonded to graphene [31]. In this case, it is assumed that the presence of covalent bonds formed between graphene and UHMWPE could slow down the thermal degradation, as stated in [39]. Moreover, the residual yields of UHMWPE/graphene nanocomposites showed a high increase due to the production of some thermally stable products as the final product of nanocomposite pyrolysis that can withstand temperatures above 700 °C [40,41]. Hence, similar to carbon fibers, this product could have two or three-dimensional structures. Related findings have also been published by other researchers [3,23,24].

**Figure 5:**
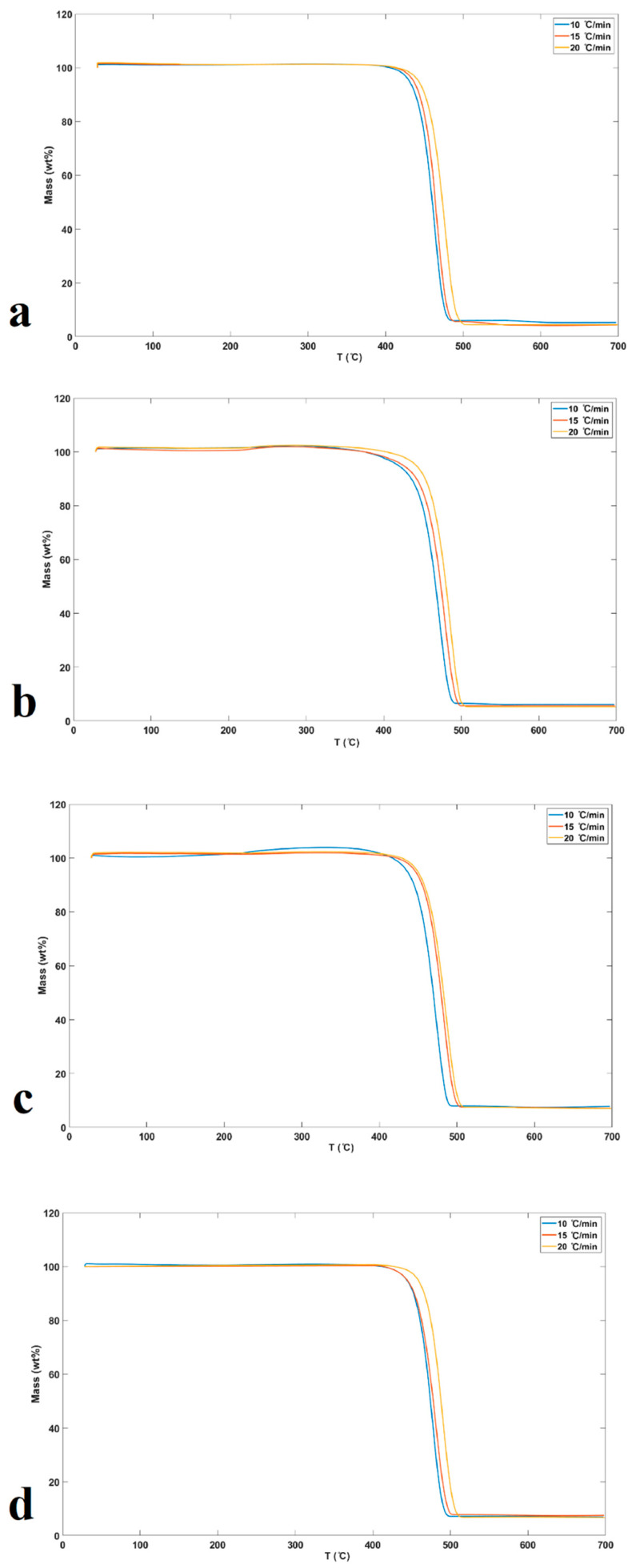
Mass loss (%) thermographs for (**a**) pure UHMWPE, (**b**) UHMWPE/graphene 0.9 wt.%, (**c**) UHMWPE/graphene 2.1 wt.%, and (**d**) UHMWPE/graphene 3.4 wt.% at heating rates of 10, 15, and 20 °C/min.

Figure 5a–d shows the TGA curves for pure UHMWPE and UHMWPE containing 0.9, 2.1, and 3.4 wt.% of graphene, respectively (under the N_2_ atmosphere at heating rates of 10, 15, and 20 °C/min) [30,42]. These figures show that as the heating rate increases, the degradation temperature is gradually shifted to a higher temperature. In general, it can be observed from Table 2 and Figure 5 that the inhibitory effect of graphene increases the thermal stability of the final nanocomposites. The thermal stability of the samples with graphene incorporation is due to the proper interaction between the graphene and the UHMWPE matrix, the shielding effect of the charred polymer on the surface, as well as the high thermal stability of graphene [26].

### 3.4. Model-Fitting Method

In this method, various models are fitted to α-temperature curves, and the E_a_ and the pre-exponential factor A are determined concurrently [21].

**Figure 6:**
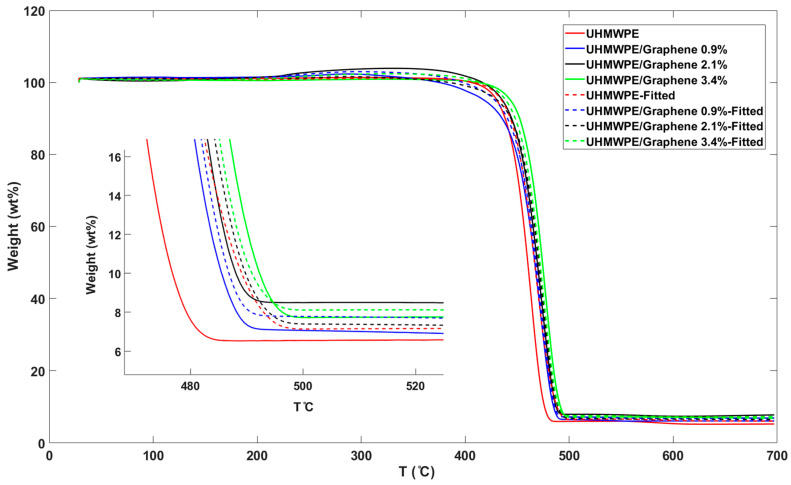
Experimental and fitted thermogravimetric analysis (TGA) curves for pure UHMWPE and UHMWPE/graphene nanocomposites at a heating rate of 10 °C/min.

When using model-fitting method, kinetic analysis is heavily reliant on the reaction model. It is also presumed that the Arrhenius type model can define the rate constant k(T) temperature dependency (does not achieve an exact distinction between the temperature-dependent k(T) and the reaction model f(α)). In addition, the reaction rate’s temperature sensitivity depends on the extent of conversion [42]. According to the F_exp_ parameter, the appropriate results were calculated using the multivariate non-linear regression method for the evaluation of the kinetic triplet for each reaction model (Figure 6). Here, in terms of fit quality, the F_exp_ is used to assess if one or more models vary statistically from the best model. The results showed that the auto-catalysis nth-order mechanism offered by f(α) = (1−α)^n^ (1 + K_cat_X) has a strong correlation with the reaction model C_n_. X is defined as the concentration of the reactant, and K_cat_ is a constant [42,43] that will properly describe experimental data with a correlation coefficient of R greater than 0.80. 

**Figure 7:**
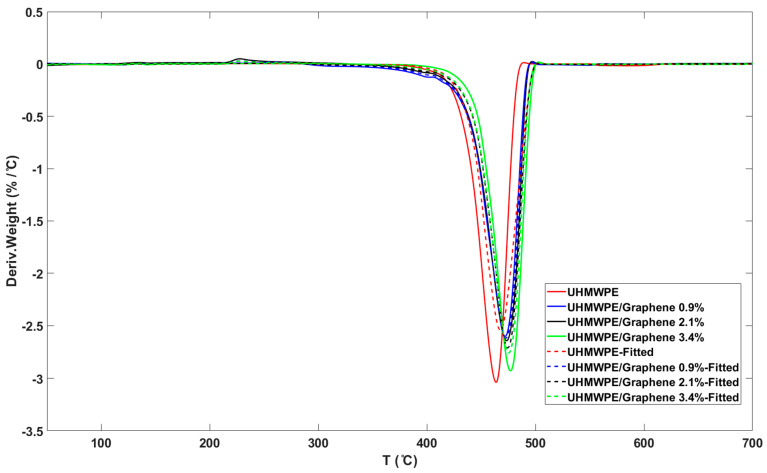
Experimental and fitted derivative thermogravimetric (DTG) curves for pure UHMWPE and UHMWPE/graphene nanocomposites at a heating rate of 10 °C/min.

Derivative thermogravimetric (DTG) are presented in Figure 7 to visualize if the model is fitting the TGA accurately by simplifying the reading of the weight versus temperature thermogram peaks as they usually occur close together. As can be seen in Figure 7, this model-fitting approach indicated some errors and the peaks in fitted curves showed considerable differences comparing to the experimental data. However, all fitted DTG curves in this model represent a similar behavior with the actual curves, showing the positive effect of incorporation of graphene nanosheets on higher thermal stability of the samples.

In Table 3, the calculated kinetic parameters are listed. It is observed that with the addition of graphene in the polymer matrix, the rate of thermal degradation decreases. This can be attributed to the improved catalytic effect that might be attributed to the presence of active functional groups on graphene and its excellent thermal conductivity [44].

### 3.5. Isoconversional Analysis

Isoconversional analysis is a “model-free” method that measures the temperatures corresponding to a permutation of heat rates, β, for all values of α without any change in the conversion function f(α) [21]. Isoconversional models offer accurate values of E_a,_ and by analyzing the reaction model (f(α)), the pre-exponential factor can be evaluated [45]. Several isoconversion methods, such as Ozawa–Flynn–Wall (OFW), Friedman, Kissinger, and Augis and Bennett, have been used to determine the E_a_ for the degradation of nanocomposites [35,46]. 

#### 3.5.1. Ozawa–Flynn–Wall (OFW) Method

Flynn, Wall, and Ozawa [47] proposed an isoconversion integral method that uses Doyle’s estimation [48] of the temperature integral. This method is based on the Equation (5):

(5) $$\mathrm{Ln}\;\beta =\mathrm{Ln}\left(\frac{\mathrm{AE}}{\mathrm{Rg}\left(\alpha \right)}\right)-5.331-1.053\left(\frac{E}{\mathrm{RT}}\right)$$

where:

(6) $$\mathrm{Ln}\left(\frac{\mathrm{AE}}{\mathrm{Rg}\left(\alpha \right)}\right)-5.331=\mathrm{const}$$

The ln(β) vs. 1/T plot obtained from the curves reported at several heating rates is a straight line, and E_a_ can be identified from the gradient’s value. If the E_a_ remains consistent with the different values of α, it indicates the presence of a single-step reaction, and a change in E_a_ value with an increase in the degree of conversion demonstrates a complex reaction mechanism [49].

**Figure 8:**
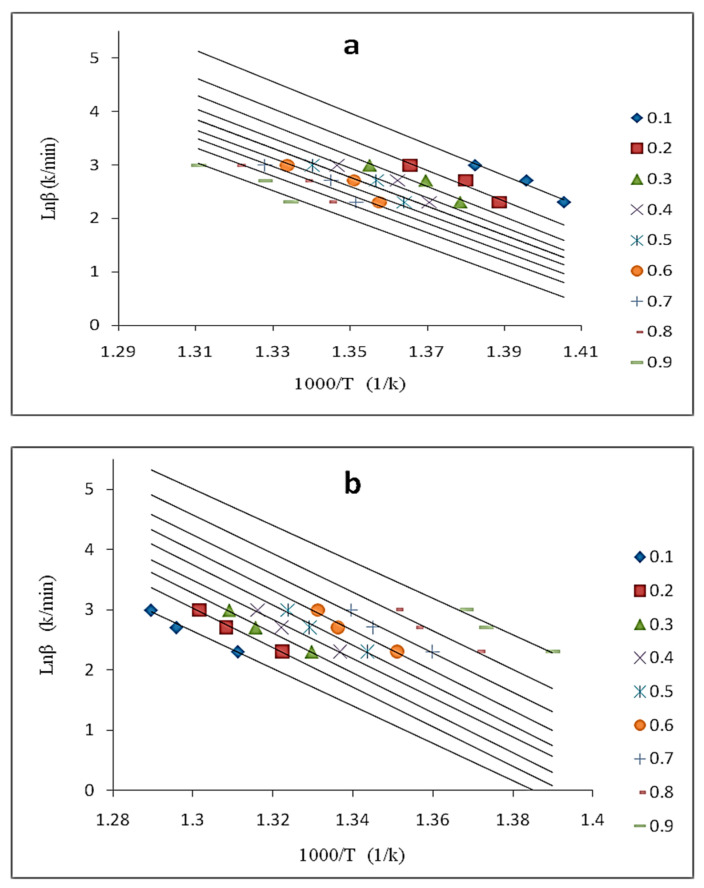
Ozawa–Flynn–Wall (OFW) plots for (**a**) neat UHMWPE, and (**b**) UHMWPE/graphene 2.1 wt.% at various fractional conversions.

Different heating rates (10, 15, and 20 °C/min) were used in this study to evaluate the “model-free” method, and the fractional conversion values ranging from 0.1 < α < 0.9 were applied to the OFW method. Figure 8a and b shows ln(β) vs. 1/T plots for the OFW method for neat UHMWPE and the sample containing 2.1 wt.% graphene, respectively. The correlation coefficient values obtained for all samples are greater or equals to 0.99, indicating that the OFW method is valid for the applied conversion range. 

**Figure 9:**
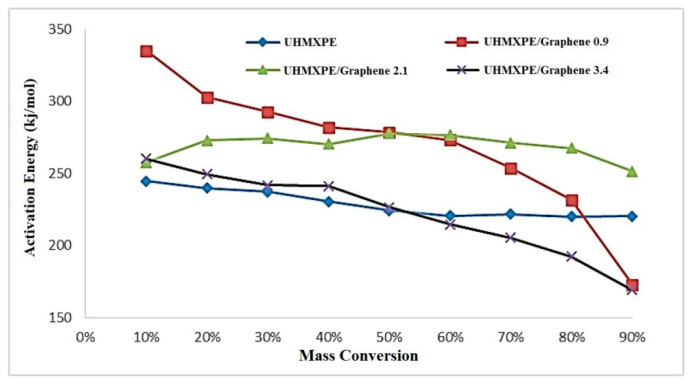
The activation energy (E_a_) vs. fractional conversion plot based on the OFW method.

Figure 9 shows the plot of E_a_ vs. fractional conversion for different samples. E_a_ of the nanocomposites containing up to 2.1 wt.% of graphene was higher than neat UHMWPE, which means that graphene’s addition decreases the thermal degradation rate of UHMWPE. This kind of behavior of nanocomposites was reported earlier [21,30].

The thermal stability of nanocomposites first increases with the added amount of graphene up to 2.1 wt.%. However, the nanocomposites with 0.9 and 3.4 wt.% showed the lowest E_a_ value with a higher degree of conversion of α = 0.9 (90%), which is less than the pure polymer. This can be due to the presence of some active groups such as hydroxyl groups, epoxy bridges, and carboxyl groups on the surface of the graphene oxide, which helps with the catalytic effects on thermal degradation [50]. Table 4 presents the average E_a_ calculated over a wide range of conversions (0.1 < α < 0.9) using this method. 

#### 3.5.2. Friedman Method

**Figure 10:**
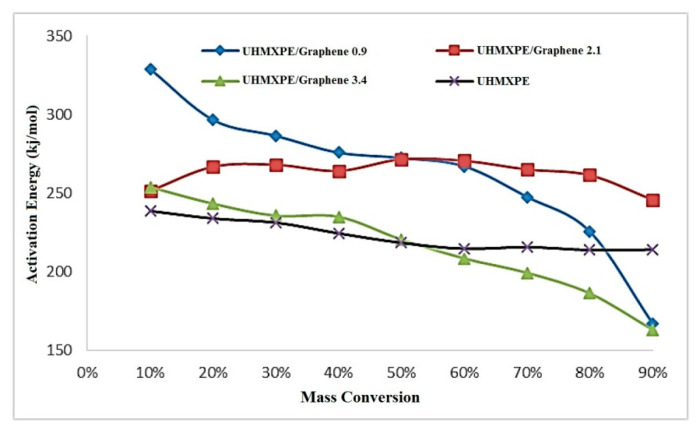
The activation energy (E_a_) vs. fractional conversion plot, calculated using the Friedman method.

Equation 7 gives the differential isoconversion method proposed by Friedman [51]:

(7) Ln[β(dαdt)]=LnA+Ln f(α)−( EaRT)

where β is the linear heating velocity (β = dT/dt), and it is a constant, α is the degree of conversion, d(α)/dt is the speed of the isothermal process, A is a pre-exponential factor or frequency factor (min^−1^), f(α) = conversion function, E_a_ is the activation energy, R is gas constant, and T is the temperature (K). The plot of ln[β(dα/dt)] vs. (1/T) is a straight line at several heating rates, and the E_a_ is measured from the slope. The equation used in the OFW method was derived from the assumption of constant E_a_, and an error can be calculated by comparison with the Friedman results by incorporating a systematic error in the measurement of E_a_ as E varies with α [21]. The E_a_ measured using the Friedman method over a wide variety of conversions (10% < α < 90%) is displayed in Figure 10 and Table 4. These values were slightly lower than those calculated by the OFW method. A systematic error could also describe the difference between the values of the Ea obtained by the two methods due to inaccurate integration.

#### 3.5.3. Kissinger Method

This method considers that at the T_m_, the maximum reaction rate occurs and assumes a constant degree of conversion (α) at this temperature [52]. The α at T_m_ varies with the heating rate (β) in some cases, and hence the precariousness is higher about putting this method into the category of isoconversion [53].

In Kissinger method, the E_a_ is calculated in constant heating rate experiments by plotting the heating rate logarithm. Without previous knowledge of the reaction order or mechanism, the E_a_ is computed, and this is the advantage of this method [54]. The Kissinger Equation is given by:

(8) $$\mathrm{Ln}\left(\frac{\beta }{T^{2}}\right)=\mathrm{Ln}\left(\frac{\mathrm{AR}}{\mathrm{Eg}\left(\alpha \right)}\right)-\frac{E}{\mathrm{RT}}$$

**Figure 11:**
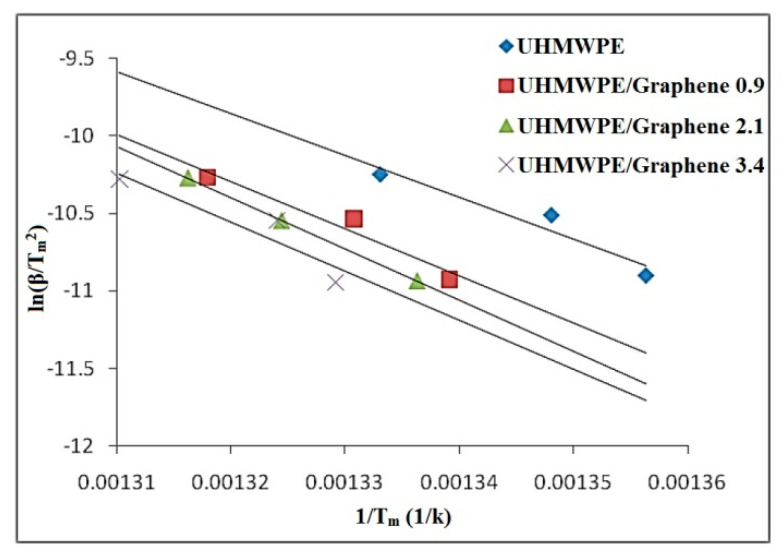
Kissinger thermal degradation plots for UHMWPE/graphene and neat UHMWPE samples.

Where T = T_m_ is the temperature that corresponds to the curvature point of the DTG peak at the thermal degradation curves and correlates to the maximum reaction rate. E_a_ values for all samples are determined from the straight-line slope of ln(β/T^2^) vs. 1/T plots with a correlation coefficient higher than 0.98, as seen in Figure 11 and Table 4.

#### 3.5.4. Augis and Bennett Method

The method proposed by Augis and Bennett (A and B) is given by Equation 9 [55]:

(9) $$\mathrm{Ln}\left(\frac{\beta }{T_{m}-T_{0}}\right)=\mathrm{LnA}-\frac{E_{a}}{{\mathrm{RT}}_{m}}$$

Where T_m_ and T_0_ are the peak temperature and the initial temperature of the DTG peak, respectively. From the slope of the straight-line ln[β/(T_m_ − T_0_)] vs. 1/T_m_ plot, E_a_ can be achieved. Like the Kissinger method, the E_a_ values determined from Augis and Bennett method are related to the peak temperature of the DTG curve. These values are equal to the values of the other isoconversion methods and are listed in Table 4.

The study of kinetic modeling and thermal degradation behavior of polymers including UHMWPE helps with better understanding of predicting thermal degradation to prevent the weaknesses of the polymeric products. As UHMWPE is an engineering polymer which is used in different industries including aerospace, the information about its thermal behavior is crucial. In addition, the kinetic studies provide information to develop pyrolysis reactors used for thermal modification of polymeric wastes [56]. By comparing the results of different kinetic methods in determination of E_a_, it can be observed that all five methods used in this study showed consistent values of E_a_ for neat UHMWPE and the samples containing 0.9 and 2.1 wt.% of graphene. However, for the sample containing 3.4 wt.% of graphene, the E_a_ values determined by Friedman and OFW methods were significantly different from the results obtained by other methods. This may be due to some existing uncertainties over baselines of the thermal analysis data or limited accuracy of determination of transformation rates [54]. Although rate-isoconversion methods like Friedman and OFW do not make any mathematical approximation, they require for the rate of transformation at temperatures that an equivalent stage of the reaction is obtained for various heating rates [54]. Accordingly, any possible errors and inaccuracies in determination of transformation rates may result in significant errors in estimation of E_a_. In addition, the obtaining results from calculation of E_a_ using different methods confirmed this fact that the presence of graphene and its interactions with the UHMWPE matrix generally increase the thermal stability and E_a_ of the polymer. However, above a specific level of graphene contents in UHMWPE matrix that the activation energy decreased is most probably due to the destructive effect of active free radicals and agglomeration effects. 

## 4. Conclusions

Thermal degradation behavior of UHMWPE/graphene nanocomposites was evaluated with different graphene contents (0.9, 2.1, and 3.4 wt.%). The prepared nanocomposite was characterized by SEM and XRD, and its thermal properties were investigated by TGA, DSC, and DTG. It is concluded from the TGA results that the thermal stability of these nanocomposites increased up to 370 °C without any significant mass loss via incorporating graphene nanosheets. Thermal degradation kinetics were investigated using model-free and model-fitting methods. The calculated E_a_ in all the methods showed a stabilizing effect on the degradation of the polymeric matrix as the graphene loading increased up to 2.1 wt.%. However, the graphene content of 3.4 wt.% or more decreased the E_a_ slightly. Modeling of degradation kinetics of UHMWPE and its nanocomposites were conducted using different methods, and the results demonstrated that although the auto-catalytic nth-order mechanism was not able to be fitted accurately with the actual TGA curves, this model could describe the degradation mechanism of UHMWPE and its nanocomposites by accurately showing the order of magnitudes of temperature thermogram peaks as well as by predicting activation energies consistent with model-free methods such as Kissinger. Furthermore, all modeling approaches in this study estimated similar values for E_a_ except the values obtained by Friedman and OFW methods for UHMWPE/graphene 3.4 wt.%, which were not close to the values obtained by other methods, which can be due to some existing uncertainties over baselines of the thermal analysis data or limited accuracy of determination of transformation rates. 

## Figures and Tables

**Table 1:** Differential scanning calorimetry (DSC) parameters for the UHMWPE and UHMWPE/graphene nanocomposites.

Sample	DH_f_ (J/g)	T_m_ (°C)
UHMWPE	123.06 (± 0.30)	137 (± 0.33)
UHMWPE/graphene 0.9 wt%	152.31 (± 0.35)	140 (± 0.32)
UHMWPE/graphene 2.1 wt%	156.95 (± 0.38)	142 (± 0.46)
UHMWPE/graphene 3.4 wt%	153.03 (± 0.51)	140 (± 0.38)

**Table 2:** Degradation temperatures of pure UHMWPE and UHMWPE/graphene nanocomposites (heating rate = 10 °C/min).

Sample	T_0.1_ (˚C)	T_0.5_ (˚C)	T_m_ (˚C)	Ash Content (%)
UHMWPE	438.3 (± 1.16)	459.9 (± 1.10)	464.1 (± 1.11)	0.6 (± 0.001)
UHMWPE/graphene 0.9 wt%	437.4 (± 1.00)	468.8 (± 1.08)	473.6 (± 1.09)	2.7 (± 0.006)
UHMWPE/graphene 2.1 wt%	446.3 (± 0.80)	471.1 (± 0.85)	475.2 (± 0.86)	3.5 (± 0.006)
UHMWPE/graphene 3.4 wt%	453.8 (± 0.27)	475.7 (± 0.28)	479.2 (± 0.29)	4.6 (± 0.002)

**Table 3:** Estimated kinetic parameter values for the nth-order model of autocatalysis.

Sample	LogA	LogK_cat_	N	E (kJ/mol)
UHMWPE	13.98	0.74	1.63	221.2
UHMWPE/graphene 0.9 wt%	14.23	0.82	1.82	249.3
UHMWPE/graphene 2.1 wt%	14.68	0.63	1.73	271.4
UHMWPE/graphene 3.4 wt%	14.43	0.31	1.75	262.7

**Table 4:** Activation energy (E_a_) values from Friedman, OFW, Kissinger, and Augis and Bennett methods.

	Friedman	OFW	Kissinger	A and B
Sample	E_a_ (kJ/mol)	E_a_ (kJ/mol)	E_a_ (kJ/mol)	E_a_ (kJ/mol)
UHMWPE	222.7	228.8	224.4	218.3
UHMWPE/graphene 0.9 wt.%	262.9	269.1	252.5	248.6
UHMWPE/graphene 2.1 wt.%	262.7	268.9	274.6	269.2
UHMWPE/graphene 3.4 wt.%	216.1	222.7	264.5	256.7

## Data Availability

The data presented in this study are available onrequest from the corresponding author.

## References

[1] Lomakin S.M., Novokshonova L.A., Brevnov P.N., Shchegolikhin A.N. (2008). Thermal properties of polyethylene/montmorillonite nanocomposites prepared by intercalative polymerization. J. Mater. Sci..

[2] Stürzel M., Kempe F., Thomann Y., Mark S., Enders M., Mülhaupt R. (2012). Novel Graphene UHMWPE Nanocomposites Prepared by Polymerization Filling Using Single-Site Catalysts Supported on Functionalized Graphene Nanosheet Dispersions. Macromolecules.

[3] Ramazani A., Saremi M.G., Amoli B.N., Izadi H. (2012). Production and characterization of UHMWPE/fumed silica nanocomposites. Polym. Compos..

[4] Das O., Neisiany R.E., Capezza A.J., Hedenqvist M.S., Försth M., Xu Q., Jiang L., Ji D., Ramakrishna S. (2020). The need for fully bio-based facemasks to counter coronavirus outbreaks: A perspective. Sci. Total Environ..

[5] Yousef S., Visco A., Galtieri G., Nocita D., Espro C. (2017). Wear behaviour of UHMWPE reinforced by carbon nanofiller and paraffin oil for joint replacement. Mater. Sci. Eng. C.

[6] Alam F., Choosri M., Gupta T.K., Varadarajan K.M., Choi D., Kumar S. (2019). Electrical, mechanical and thermal properties of graphene nanoplatelets reinforced UHMWPE nanocomposites. Mater. Sci. Eng. B.

[7] Martínez-Morlanes M.J., Pascual F.J., Guerin G., Puértolas J.A. (2021). Influence of processing conditions on microstructural, mechanical and tribological properties of graphene nanoplatelet reinforced UHMWPE. J. Mech. Behav. Biomed. Mater..

[8] Grinev V.G., Krasheninnikov V.G., Zabolotnov A.S., Ladygina T.A., Brevnov P.N., Novokshonova L.A., Berlin A.A. (2018). The Effect of Filler Type on the Mechanical Properties of Composite Materials Based on Ultra-High-Molecular-Weight Polyethylene. Polym. Sci. Ser. D.

[9] Wang B., Li H., Li L., Chen P., Wang Z., Gu Q. (2013). Electrostatic adsorption method for preparing electrically conducting ultrahigh molecular weight polyethylene/graphene nanosheets composites with a segregated network. Compos. Sci. Technol..

[10] Rezvani Ghomi E., Esmaeely Neisiany R., Nouri Khorasani S., Dinari M., Ataei S., Koochaki M.S., Ramakrishna S. (2020). Development of an epoxy self-healing coating through the incorporation of acrylic acid-co-acrylamide copolymeric gel. Prog. Org. Coat..

[11] Ghomi E.R., Khorasani S.N., Kichi M.K., Dinari M., Ataei S., Enayati M.H., Koochaki M.S., Neisiany R.E. (2020). Synthesis and characterization of TiO2/acrylic acid-co-2-acrylamido-2-methyl propane sulfonic acid nanogel composite and investigation its self-healing performance in the epoxy coatings. Colloid Polym. Sci..

[12] Kaminsky W., Funck A., Klinke C. (2008). In-situ Polymerization of Olefins on Nanoparticles or Fibers by Metallocene Catalysts. Top. Catal..

[13] Kaminsky W., Funck A., Wiemann K. (2006). Nanocomposites by In Situ Polymerization of Olefins with Metallocene Catalysts. Macromol. Symp..

[14] Thongruang W., Balik C.M., Spontak R.J. (2002). Volume-exclusion effects in polyethylene blends filled with carbon black, graphite, or carbon fiber. J. Polym. Sci. Part B Polym. Phys..

[15] Zhu Y., Murali S., Cai W., Li X., Suk J.W., Potts J.R., Ruoff R.S. (2010). Graphene and Graphene Oxide: Synthesis, Properties, and Applications. Adv. Mater..

[16] Hu W., Zhan J., Wang X., Hong N., Wang B., Song L., Stec A.A., Hull T.R., Wang J., Hu Y. (2014). Effect of Functionalized Graphene Oxide with Hyper-Branched Flame Retardant on Flammability and Thermal Stability of Cross-Linked Polyethylene. Ind. Eng. Chem. Res..

[17] Khosravi F., Nouri Khorasani S., Khalili S., Esmaeely Neisiany R., Rezvani Ghomi E., Ejeian F., Das O., Nasr-Esfahani M.H. (2020). Development of a Highly Proliferated Bilayer Coating on 316L Stainless Steel Implants. Polymers.

[18] Das O., Hedenqvist M.S., Johansson E., Olsson R.T., Loho T.A., Capezza A.J., Singh Raman R.K., Holder S. (2019). An all-gluten biocomposite: Comparisons with carbon black and pine char composites. Compos. Part A Appl. Sci. Manuf..

[19] Das O., Loho T.A., Capezza A.J., Lemrhari I., Hedenqvist M.S. (2018). A Novel Way of Adhering PET onto Protein (Wheat Gluten) Plastics to Impart Water Resistance. Coatings.

[20] Khosravi F., Nouri Khorasani S., Rezvani Ghomi E., Kichi M.K., Zilouei H., Farhadian M., Esmaeely Neisiany R. (2019). A bilayer GO/nanofibrous biocomposite coating to enhance 316L stainless steel corrosion performance. Mater. Res. Express.

[21] Shariati J., Saadatabadi A.R., Khorasheh F. (2012). Thermal Degradation Behavior and Kinetic Analysis of Ultra High Molecular Weight Polyethylene Based Multi-Walled Carbon Nanotube Nanocomposites Prepared Via in-situ Polymerization. J. Macromol. Sci. Part A.

[22] Kuilla T., Bhadra S., Yao D., Kim N.H., Bose S., Lee J.H. (2010). Recent advances in graphene based polymer composites. Prog. Polym. Sci..

[23] Quan H., Zhang B.-q., Zhao Q., Yuen R.K.K., Li R.K.Y. (2009). Facile preparation and thermal degradation studies of graphite nanoplatelets (GNPs) filled thermoplastic polyurethane (TPU) nanocomposites. Compos. Part A Appl. Sci. Manuf..

[24] Vadukumpully S., Paul J., Mahanta N., Valiyaveettil S. (2011). Flexible conductive graphene/poly(vinyl chloride) composite thin films with high mechanical strength and thermal stability. Carbon.

[25] Rezvani Ghomi E., Khosravi F., Mossayebi Z., Saedi Ardahaei A., Morshedi Dehaghi F., Khorasani M., Neisiany R.E., Das O., Marani A., Mensah R.A. (2020). The Flame Retardancy of Polyethylene Composites: From Fundamental Concepts to Nanocomposites. Molecules.

[26] Shakiba M., Kakoei A., Jafari I., Rezvani Ghomi E., Kalaee M., Zarei D., Abdouss M., Shafiei-Navid S., Khosravi F., Ramakrishna S. (2021). Kinetic Modeling and Degradation Study of Liquid Polysulfide Resin-Clay Nanocomposite. Molecules.

[27] Kim J.Y., Park H.S., Kim S.H. (2007). Multiwall-carbon-nanotube-reinforced poly(ethylene terephthalate) nanocomposites by melt compounding. J. Appl. Polym. Sci..

[28] Stürzel M., Thomann Y., Enders M., Mülhaupt R. (2014). Graphene-Supported Dual-Site Catalysts for Preparing Self-Reinforcing Polyethylene Reactor Blends Containing UHMWPE Nanoplatelets and in Situ UHMWPE Shish-Kebab Nanofibers. Macromolecules.

[29] Nikkhah S.J., Ramazani S.A., Baniasadi H., Tavakolzadeh F. (2009). Investigation of properties of polyethylene/clay nanocomposites prepared by new in situ Ziegler–Natta catalyst. Mater. Des..

[30] Chrissafis K., Bikiaris D. (2011). Can nanoparticles really enhance thermal stability of polymers? Part I: An overview on thermal decomposition of addition polymers. Thermochim. Acta.

[31] Shafiee M., Ramazani S.A. (2014). Preparation and Characterization of UHMWPE/Graphene Nanocomposites Using Bi-Supported Ziegler-Natta Polymerization. Int. J. Polym. Mater. Polym. Biomater..

[32] Shakiba M., Nabavi S.R., Emadi H., Faraji M. (2021). Development of a superhydrophilic nanofiber membrane for oil/water emulsion separation via modification of polyacrylonitrile/polyaniline composite. Polym. Adv. Technol..

[33] Zaaba N.I., Foo K.L., Hashim U., Tan S.J., Liu W.-W., Voon C.H. (2017). Synthesis of Graphene Oxide using Modified Hummers Method: Solvent Influence. Procedia Eng..

[34] Chrissafis K., Paraskevopoulos K.M., Tsiaoussis I., Bikiaris D. (2009). Comparative study of the effect of different nanoparticles on the mechanical properties, permeability, and thermal degradation mechanism of HDPE. J. Appl. Polym. Sci..

[35] Chrissafis K. (2009). Kinetics of thermal degradation of polymers. J. Therm. Anal. Calorim..

[36] Mittal V., Luckachan G.E., Matsko N.B. (2014). PE/Chlorinated-PE Blends and PE/Chlorinated-PE/Graphene Oxide Nanocomposites: Morphology, Phase Miscibility, and Interfacial Interactions. Macromol. Chem. Phys..

[37] Haddadi S.A., Saadatabadi A.R., Kheradmand A., Amini M., Ramezanzadeh M. (2019). SiO2-covered graphene oxide nanohybrids for in situ preparation of UHMWPE/GO(SiO2) nanocomposites with superior mechanical and tribological properties. J. Appl. Polym. Sci..

[38] Das O., Kim N.K., Hedenqvist M.S., Lin R.J.T., Sarmah A.K., Bhattacharyya D. (2018). An Attempt to Find a Suitable Biomass for Biochar-Based Polypropylene Biocomposites. Environ. Manag..

[39] Wang K., Liu M., Song C., Shen L., Chen P., Xu S. (2018). Surface-conductive UHMWPE fibres via in situ reduction and deposition of graphene oxide. Mater. Des..

[40] Sun T., Luo W., Luo Y., Wang Y., Zhou S., Liang M., Chen Y., Zou H. (2020). Self-Reinforced Polypropylene/Graphene Composite with Segregated Structures To Achieve Balanced Electrical and Mechanical Properties. Ind. Eng. Chem. Res..

[41] Das O., Sarmah A.K. (2015). Value added liquid products from waste biomass pyrolysis using pretreatments. Sci. Total Environ..

[42] Chrissafis K., Paraskevopoulos K.M., Pavlidou E., Bikiaris D. (2009). Thermal degradation mechanism of HDPE nanocomposites containing fumed silica nanoparticles. Thermochim. Acta.

[43] Lomakin S.M., Dubnikova I.L., Shchegolikhin A.N., Zaikov G.E., Kozlowski R., Kim G.M., Michler G.H. (2008). Thermal degradation and combustion behavior of the polyethylene/clay nanocomposite prepared by melt intercalation. J. Therm. Anal. Calorim..

[44] Tan L., Xu J., Zhang X., Hang Z., Jia Y., Wang S. (2015). Synthesis of g-C3N4/CeO2 nanocomposites with improved catalytic activity on the thermal decomposition of ammonium perchlorate. Appl. Surf. Sci..

[45] Farjas J., Roura P. (2011). Isoconversional analysis of solid state transformations. J. Therm. Anal. Calorim. J. Anal. Calorim.

[46] Antoniadis G., Paraskevopoulos K.M., Bikiaris D., Chrissafis K. (2009). Melt-crystallization mechanism of poly(ethylene terephthalate)/multi-walled carbon nanotubes prepared by in situ polymerization. J. Polym. Sci. Part B Polym. Phys..

[47] Ozawa T. (2000). Thermal analysis—review and prospect. Thermochim. Acta.

[48] Doyle C.D. (1961). Kinetic analysis of thermogravimetric data. J. Appl. Polym. Sci..

[49] Opfermann J.R., Kaisersberger E., Flammersheim H.J. (2002). Model-free analysis of thermoanalytical data-advantages and limitations. Thermochim. Acta.

[50] Król-Morkisz K., Pielichowska K., Pielichowski K., Majka T.M. (2019). 13-Thermal Decomposition of Polymer Nanocomposites With Functionalized Nanoparticles. Polymer Composites with Functionalized Nanoparticles.

[51] Friedman H.L. (1969). New methods for evaluating kinetic parameters from thermal analysis data. J. Polym. Sci. Part B Polym. Lett..

[52] Fernandez A., Mazza G., Rodriguez R. (2018). Thermal decomposition under oxidative atmosphere of lignocellulosic wastes: Different kinetic methods application. J. Environ. Chem. Eng..

[53] Kissinger H.E. (1957). Reaction Kinetics in Differential Thermal Analysis. Anal. Chem..

[54] Starink M.J. (2003). The determination of activation energy from linear heating rate experiments: A comparison of the accuracy of isoconversion methods. Thermochim. Acta.

[55] Boswell P.G. (1980). On the calculation of activation energies using a modified Kissinger method. J. Therm. Anal. Calorim..

[56] Ray S., Cooney R.P., Kutz M. (2018). Chapter 9—Thermal Degradation of Polymer and Polymer Composites. Handbook of Environmental Degradation of Materials (Third Edition).

